# Association between blood essential metal elements in early pregnancy and gestational diabetes mellitus

**DOI:** 10.3389/fnut.2025.1554840

**Published:** 2025-06-19

**Authors:** Guozhen Chen, Li Wu, Cunwei Ji, Jianhong Xia, Guocheng Liu

**Affiliations:** Guangdong Women and Children Hospital, Guangzhou, China

**Keywords:** essential metal elements, gestational diabetes mellitus, inflammatory indicators, mixed exposure, mediation

## Abstract

**Objectives:**

The purpose was to assess the levels of iron (Fe), magnesium (Mg), copper (Cu), calcium (Ca), zinc (Zn) in the blood of pregnant women during early pregnancy, and to evaluate their potential association with gestational diabetes mellitus (GDM).

**Methods:**

We enrolled 9,112 pregnant women who underwent testing for essential metal elements at Guangdong Women and Children Hospital during the first trimester in 2015–2022. The basic information of pregnant women and peripheral blood samples were collected, and five essential metal elements in whole blood were detected by atomic absorption spectrometry. The relationship between these essential metal elements and GDM was analyzed using the generalized linear regression model (GLM), weighted quantile sum regression (WQS), quantile g-computation regression (QGC), and Bayesian kernel machine regression (BKMR).

**Results:**

Analysis of the correlation between essential metal elements and GDM revealed significant associations. Compared with the first quantile concentration level, the fourth quantile level of Fe (*OR* = 1.347, 95%*CI*: 1.158 ~ 1.568), Zn (*OR* = 1.379, 95%*CI*: 1.185 ~ 1.606) and Mg (*OR* = 1.192, 95%*CI*: 1.022 ~ 1.392) exhibited significant associations with GDM. Restricted cubic spline (RCS) analysis showed a positive linear relationship between Fe, Zn, and Mg and GDM risk (*P_overall_* < 0.05 and *P_non-linear_* > 0.05). WQS analysis showed that the WQS index had a significant positive correlation with GDM (*OR* = 1.129, 95%*CI*: 1.023 ~ 1.247), with Fe (0.446) having greater weight. QGC analysis revealed a positive correlation between the combined action of five essential metal elements and GDM risk (*OR* = 1.161, 95%*CI*: 1.075 ~ 1.248), with Zn (0.454) and Fe (0.417) showing greater influence. In BKMR analysis, the combined effect of all essential metal elements on GDM showed an overall upward trend, with Fe (PIP = 0.772) having the most significant influence. No interaction between essential metal elements and GDM was found in this study.

**Conclusion:**

Higher levels of Fe, Zn and Mg were positively correlated with GDM risk. The combined action of five essential metal elements was positively correlated with GDM, with Fe identified as playing the most significant role.

## Highlights

- The aim of this study was to evaluate the levels of iron (Fe), magnesium (Mg), copper (Cu), calcium (Ca), and zinc (Zn) in the blood of pregnant women during early pregnancy and to explore their individual, combined, and interactive effects with gestational diabetes mellitus (GDM).
- This prospective cohort study enrolled 9,112 pregnant women who underwent testing for essential trace elements at Guangdong Women and Children Hospital during the first trimester between 2015 and 2022.
- The study found that higher levels of Fe, Zn, and Mg were positively correlated with the risk of GDM, highlighting the importance of maintaining appropriate levels of essential trace elements in pregnant women during early pregnancy. Furthermore, the research revealed a positive correlation between the combined exposure to essential trace elements and GDM risk, with Fe playing a predominant role.

## 1 Introduction

Gestational diabetes mellitus (GDM) was defined as diabetes mellitus that develops during pregnancy in women who previously had normal glucose metabolism, representing one of the most prevalent complications during gestation (1). In recent years, the global prevalence of GDM has been rising due to increasing rates of overweight and obesity among women of childbearing age, as well as an aging population (2, 3). Numerous studies have highlighted the short-term and long-term adverse health effects of GDM on both pregnant women and their offspring (4). GDM not only contributes to adverse pregnancy outcomes such as premature delivery, dystocia, and macrosomia, but also elevates the risk of future cardiovascular and metabolic diseases (5–7). Moreover, maternal hyperglycemia during early pregnancy epigenetically programmed offspring’s metabolic health, as highlighted in the DOHaD paradigm (8, 9). At present, the etiology of GDM was not clear, which brought difficulties to clinical diagnosis and treatment.

The common risk factors for GDM included genetic predisposition, unhealthy lifestyle, and social factors (10–12). Some researchers also found that intestinal flora imbalance caused diabetes through inflammation, insulin resistance, and nutritional signals (13, 14). In addition, increasing animal and metabolic studies demonstrated that the steady-state imbalance of essential metal elements including iron (Fe), calcium (Ca), zinc (Zn), copper (Cu) and magnesium (Mg) was also an important risk factor leading to GDM (15, 16). Among these biological samples, blood levels of essential metal elements were relatively stable (17, 18). Therefore, whole blood samples were often used to monitor the levels of these five essential metals to assess the health status of pregnant women (19). They were also used to intervene in the dietary supplementation, so as to maintain the normal physiological and metabolic functions of pregnant women (20).

The deficiency or excess of essential metal elements in pregnant women may be associated with inflammation and oxidative stress, influencing glucose metabolism and insulin sensitivity and thereby increasing the risk of GDM (19). Studies have found that oxidative stress mediated by Fe overload may be related to the occurrence of gestational diabetes (21, 22). In a prospective study of pregnant women, the high level of plasma Cu in early pregnancy was positively correlated with the glucose level in the middle and late pregnancy (23). Zhao et al. (24) also found that Impairment of β-cells by Cu homeostasis is related to the occurrence of GDM. In addition, more and more studies showed that other essential metal elements such as Zn and Mg were also related to GDM (25). A retrospective cohort study discovered that lower Ca and Mg concentrations within a certain range before 24 weeks’ gestation might prospectively impair fasting plasma glucose levels during pregnancy (26). However, the research on the relationship between essential metal elements and GDM was limited, and the research results were still inconsistent (27, 28). Variations in sample sizes and research methodologies may compromise the consistency of conclusions drawn from these studies (27–29).

Furthermore, most current research focused on the impact of individual essential metal elements on GDM (30–33), overlooking the simultaneous consumption and potential interactions among multiple essential metals that pregnant women typically experience (34). Few studies explored the combined effects of various essential metal elements on GDM (35). Previous studies suggested that it was necessary to carry out related research in pregnant women to verify the association between essential metal elements and GDM, so as to identify and intervene GDM-related risk factors early.

Therefore, we hypothesize that there was a correlation between the levels of essential metal elements during early pregnancy and GDM. This study aimed to assess the levels of five essential metal elements in the blood of pregnant women in the first trimester and explore their individual associations with GDM. Given that pregnant women are exposed to multiple essential metal elements simultaneously in the first trimester, using a combined exposure model, the relationship between the joint action and interaction of essential metal elements and GDM was analyzed.

## 2 Materials and methods

### 2.1 Study design and participants

This prospective cohort study enrolled 9,801 pregnant women who met inclusion criteria between January 2015 and December 2022. Participants were included after completing baseline data collection during their initial pregnancy examination, with follow-up conducted through the second and third trimesters for oral glucose tolerance tests (OGTT) in outpatient settings. Following application of exclusion criteria, 9,112 participants remained for analysis, based on their baseline and follow-up survey data. The study received approval from the Ethics Committee of Guangdong Women and Children Hospital (ID: 202401082).

Inclusion criteria: (1) Age 18 ~ 45 years; (2) Singleton pregnancy; (3) Being a Guangdong resident planning to deliver at the study hospital; (4) Detection of blood essential metal elements in early pregnancy (<14 weeks). Exclusion criteria: (1) Pre-pregnancy patients complicated with metabolic diseases such as diabetes and severe organ diseases; (2) Abortion and induced labor during pregnancy; (3) Suffering from other serious medical diseases during pregnancy; (4) Incomplete clinical information.

**Figure 1:**
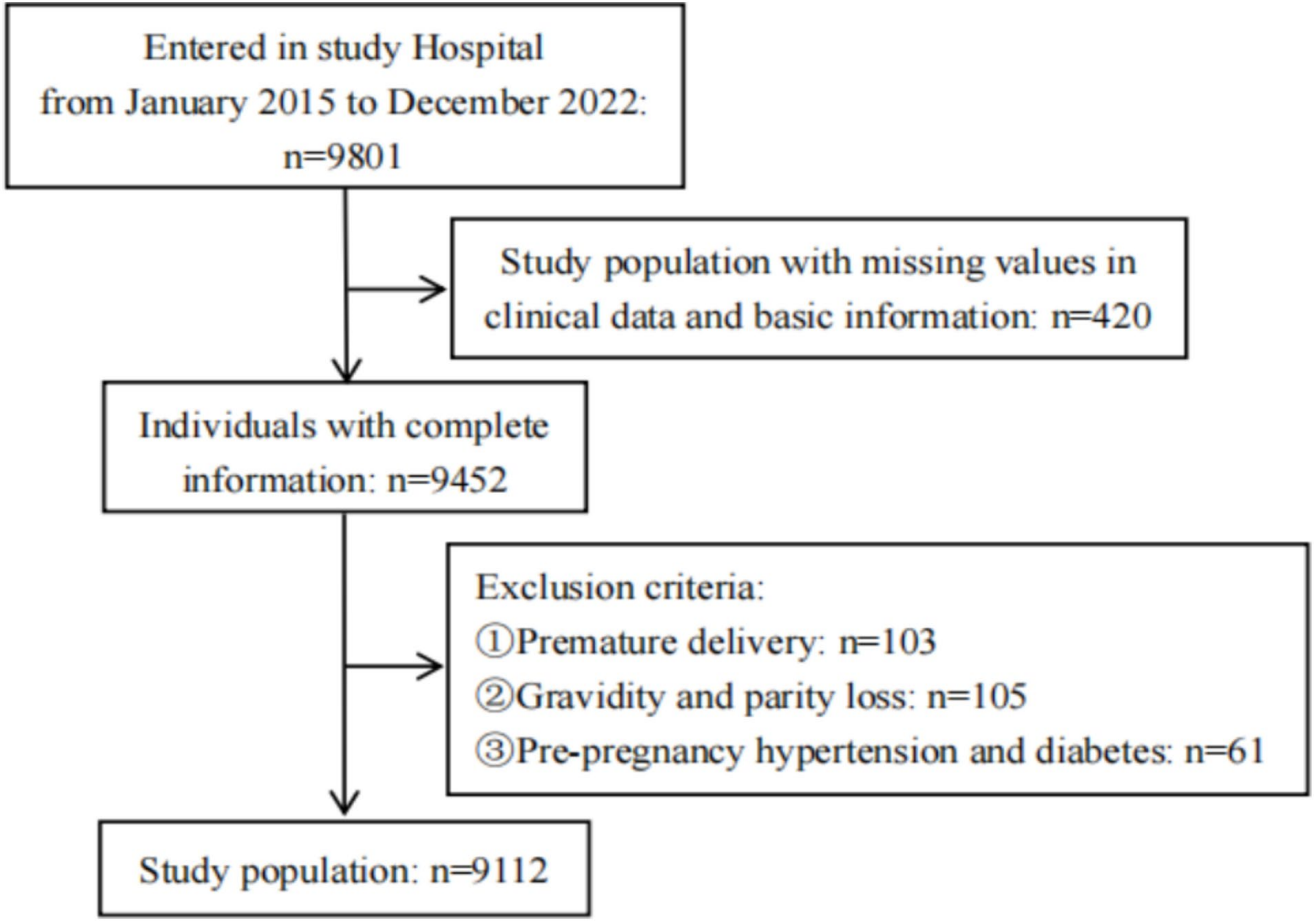
Flowchart of study population.

This study performed a sample size estimation based on a cohort study design. With *α* = 0.05 and *β* = 0.10, the calculated required sample size was 3,870. Considering potential issues such as loss to follow-up, the sample size was increased by 20%. The final sample size included in this study meets the required criteria (Figure 1).

### 2.2 Definitions

In this study, GDM was taken as the outcome index, and the diagnosis information was obtained from the electronic medical record system. Diagnostic criteria of GDM: All pregnant women who have not been diagnosed with pre-pregnancy diabetes or gestational diabetes were given 75 g OGTT at the first visit after 24–28 weeks of pregnancy. The diagnostic thresholds for the 75 g OGTT were as follows: Fasting plasma glucose (FPG) < 5.1 mmol/L, or 1-h plasma glucose (1hPG) < 10.0 mmol/L, or 2-h plasma glucose (2hPG) < 8.5 mmol/L (36).

### 2.3 Basic data collection

During the initial pregnancy examination, basic information including the age, pre-pregnancy height and weight, last menstrual period, maternity history, and medical history of the pregnant women was collected. This information was self-reported and recorded in the electronic medical record system, which was queried for data retrieval. Pre-pregnancy BMI was calculated by dividing self-reported pre-pregnancy weight by the square of measured height (37).

### 2.4 Measurement of trace elements

Whole blood samples were collected from pregnant women in the first trimester (12.27 ± 0.9 weeks) by trained nurses. Two mL venous blood was collected with EDTA-K2 vacuum anticoagulant tubes from the antecubital region. Samples were transported at room temperature and refrigerated at 4°C, and analyzed within 3 days of collection.

The flame atomic absorption spectrometer (BH7100S), calibration solution, and quality control materials were sourced from Beijing Bohui Innovation Biotechnology Co., Ltd. The instrument utilized gas flow control technology in the tungsten boat metal furnace. Prior to testing, whole blood samples were thoroughly mixed using a vortex for at least 1 min. Subsequently, 40 μL of the whole blood sample was diluted with 1.96 mL of diluent. After mixing, the elemental concentrations in all blood samples were measured via flame atomic absorption spectrometry within 2 h. The instrument and element lamp were preheated for 30 min to ensure baseline stability without ignition during this period.

### 2.5 Quality control

Participants were selected in strict accordance with the inclusion and exclusion criteria. Data integrity was maintained by carefully cleaning and verifying participant information and laboratory results. Covariates were included in all statistical analysis models, and sensitivity analysis was conducted to evaluate the reliability of the results.

Biological samples were collected following a standardized protocol, and essential metal elements were measured using a unified method. To ensure accuracy and precision, regular calibration and quality control were performed. Before testing each batch of samples, a standard curve along with low and high-level quality control samples were conducted. The quality control results for each batch met the requirement of a standard deviation within twice the target value. For the five essential metal elements, the baseline stability was maintained within 0.005 Abs. Instrument sensitivity was confirmed by spraying the sensitivity standard detection solution, with the characteristic concentrations for each metal element as follows: Cu 0.035 mg/L/1%, Zn 0.015 mg/L/1%, Ca 0.080 mg/L/1%, Mg 0.040 mg/L/1%, and Fe 1.05 mg/L/1%.

To address potential batch effects due to the extended collection period, only data from pregnant women’s first visits were used. Consistency in experimental equipment, measurement protocols, and procedures was maintained through regular calibration and maintenance to ensure instrument stability. The standard deviation for each batch was set to 2 times the target value, keeping batch-to-batch variability within an acceptable range. During the experimental design stage, potential sources of variation were strictly controlled, and standardized preprocessing steps were applied. Additionally, principal component analysis (PCA) was used to detect batch effects, and methods were applied, when necessary, to correct residual batch-related variability, ensuring result reliability.

### 2.6 Statistical analysis

In this study, all data analyses were performed using R version 4.3.1 software. Assessment of statistical significance was based on two-tailed *p*-values, with a *p* < 0.05 threshold considered to be statistically significant. No missing data were included in the analysis, as cases with missing values were excluded during participant selection. In descriptive analysis, classified variables were characterized by frequency and composition ratio, while continuous variables were presented as mean ± standard deviation (SD). Independent sample *t*-tests were used to compare two groups of continuous variables assumed to follow a normal distribution, and *χ*^2^ tests were utilized for comparing constituent ratios. Normality tests confirmed that concentrations of five essential metal elements in pregnant women’s blood approximated a normal distribution. Pearson correlation analysis was conducted to examine associations between these essential metal elements.

The study utilized generalized linear regression (GLM) evaluate the relationship between a single essential metal element and GDM. According to the quartile of five essential metal elements, the subjects were divided into four groups, and the risk of GDM in other quantile levels before and after adjusting covariate was estimated with the first quantile level as reference. The median level of the quartile array of each essential metal element was included in the model for trend test (*P* for trend), and then the trend test is corrected by using the false discovery rate (FDR) to control the false positive rate (38). To explore the dose–response relationship, restricted cubic splines (RCS) were employed for visual analysis, setting nodes at the 10th, 50th, and 90th percentiles with the 50th percentile serving as the reference value. Both overall correlation (*P*_overall_) and non-linear correlation (*P*_non-linear_) were found to be less than 0.05, indicating a non-linear dose–response relationship. A *P*_overall_ < 0.05 with *P*_non-linear_ > 0.05 suggested a linear dose–response relationship (39).

Bayesian kernel machine regression can analyze the complexity of the combined action of chemicals. This study primarily aimed to evaluate the combined effect of five essential metal elements in blood on GDM, identify the essential metal elements that significantly impact the outcome, fit the dose–response relationship of this combined effect, and explore potential interactions between these essential metal elements (40). The relative importance of each essential metal element was determined by calculating the posterior inclusion probability (PIP). The closer the PIP value is to 1, the greater the role of the essential metal element in the joint effect. In this study, BKMR model was run for 25,000 iterations. The r package used was “bkmr.”

In order to verify the robustness of our main results, two sensitivity analyses were performed. Firstly, weighted quantile sum regression (WQS) and quantile g-computation regression (QGC) were used to verify the results of mixed exposure analysis. Both models are classical mixed exposure models (41). The overall effect of the combined action of essential metal elements on GDM was estimated. Additionally, the contribution of each individual essential metal element was analyzed and displayed. The R-package used were “gWQS” and “qgcomp.” Secondly, in order to verify the interaction between essential metal elements in the results of BKMR model, the essential metal elements are divided into two levels according to the median. Evaluation of multiplication terms in Logistic regression analysis of multiplication interaction. The additive interaction was evaluated by using R-package “epiR” and three indicators: relative excess risk due to interaction (RERI), attributable promotion due to interaction (AP) and synergy index (S). If 95%CI of RERI and AP index does not contain 0 and 95%CI of S index does not contain 1, it is considered that there is additive interaction between essential metal elements, in which RERI value and AP value > 0 are synergistic, RERI < 0 and AP < 0 are antagonistic, *S* value > 1 is synergistic and *S* < 1 is antagonistic (42).

To improve clarity, we have specified the covariates included in each model (GLM, BKMR, WQS, QGC). All models included the following covariates: maternal age in years at enrollment (continuous) (43), pre-pregnancy BMI (kg/m^2^, continuous) (44), parity (45), season (categorized as spring, summer, autumn, winter) (46–48) and gestational week (weeks, continuous) (49) at blood specimen collection. Because factors such as gestational week and season may affect the content of essential metal elements, attention should be paid to these variables (50).

## 3 Results

### 3.1 Characteristics of the study population

The population characteristics of the pregnant women recruited in our study were summarized in Table 1. The average age of 9,112 pregnant women was 30.47 ± 4.37 years old, and the average BMI before pregnancy was 20.79 ± 2.68 kg/m^2^. Of these women, 18.62% (1,697/9112) pregnant women were subsequently diagnosed with GDM. The age, parity, gravidity and pre-pregnancy BMI of pregnant women with GDM were higher than those of pregnant women without GDM (*p* < 0.001).

**Table 1:** Demographic characteristics of research objects (*N* = 9,112).

Variables	GDM (*n* = 1,697)	Non-GDM (*n* = 7,415)	*t/χ^2^*	*P*
Maternal age (years)	32.04 ± 4.43	30.14 ± 4.29	**16.041**	**<0.001**
18 ~ 25	43 (2.53)	575 (7.75)	**215.610**	**<0.001**
26 ~ 30	606 (35.71)	3,657 (49.32)		
31 ~ 45	1,048 (61.76)	3,183 (42.93)		
Pre-pregnancy BMI (kg/m^2^)	21.56 ± 2.92	20.63 ± 2.60	**12.046**	**<0.001**
<18.5	233 (13.73)	1,515 (20.43)	**117.100**	**<0.001**
18.5 ~ 23.9	1,127 (66.41)	5,079 (68.50)		
≥24.0	337 (19.86)	821 (11.07)		
Parity				
Primiparity	821 (48.38)	4,013 (54.12)	**18.041**	**<0.001**
Multiparity	876 (51.62)	3,402 (45.88)		
Gravidity (times)				
1	526 (31.00)	2,844 (38.35)	**31.772**	**<0.001**
≥2	1,171 (69.00)	4,571 (61.65)		

Continuous variables in the table are represented by −*x* ± *s*, and classified variables are represented by *n* (%). Bold black font indicates statistically significant differences between groups (*P* < 0.05).

### 3.2 Element concentrations in whole blood

The characteristics of essential metal elements in blood of pregnant women in the first trimester were shown in Table 2. Compared with the non-GDM group, the concentration levels of essential metal elements such as Fe (*p* < 0.001), Zn (*p* < 0.001), Cu (*p* = 0.008) and Mg (*p* < 0.001) in GDM group were higher.

**Table 2:** Characteristics of essential metal elements in pregnant women’s blood (*N* = 9,112).

Variables	GDM (*n* = 1,697)	Non-GDM (*n* = 7,415)	*t*	*P*
Fe (mmol/L)	7.83 ± 0.68	7.74 ± 0.67	**4.639**	**< 0.001**
Ca (mmol/L)	1.55 ± 0.10	1.55 ± 0.10	1.318	0.187
Zn (μmol/L)	85.82 ± 12.37	84.29 ± 12.02	**4.633**	**< 0.001**
Cu (μmol/L)	23.06 ± 4.65	22.73 ± 4.5	**2.651**	**0.008**
Mg (mmol/L)	1.40 ± 0.13	1.38 ± 0.13	**4.409**	**< 0.001**

Continuous variables in the table are represented by −*x* ± *s*. Bold black font indicates statistically significant differences between groups (*P* < 0.05).

### 3.3 Correlation analysis between essential metal elements

**Figure 2:**
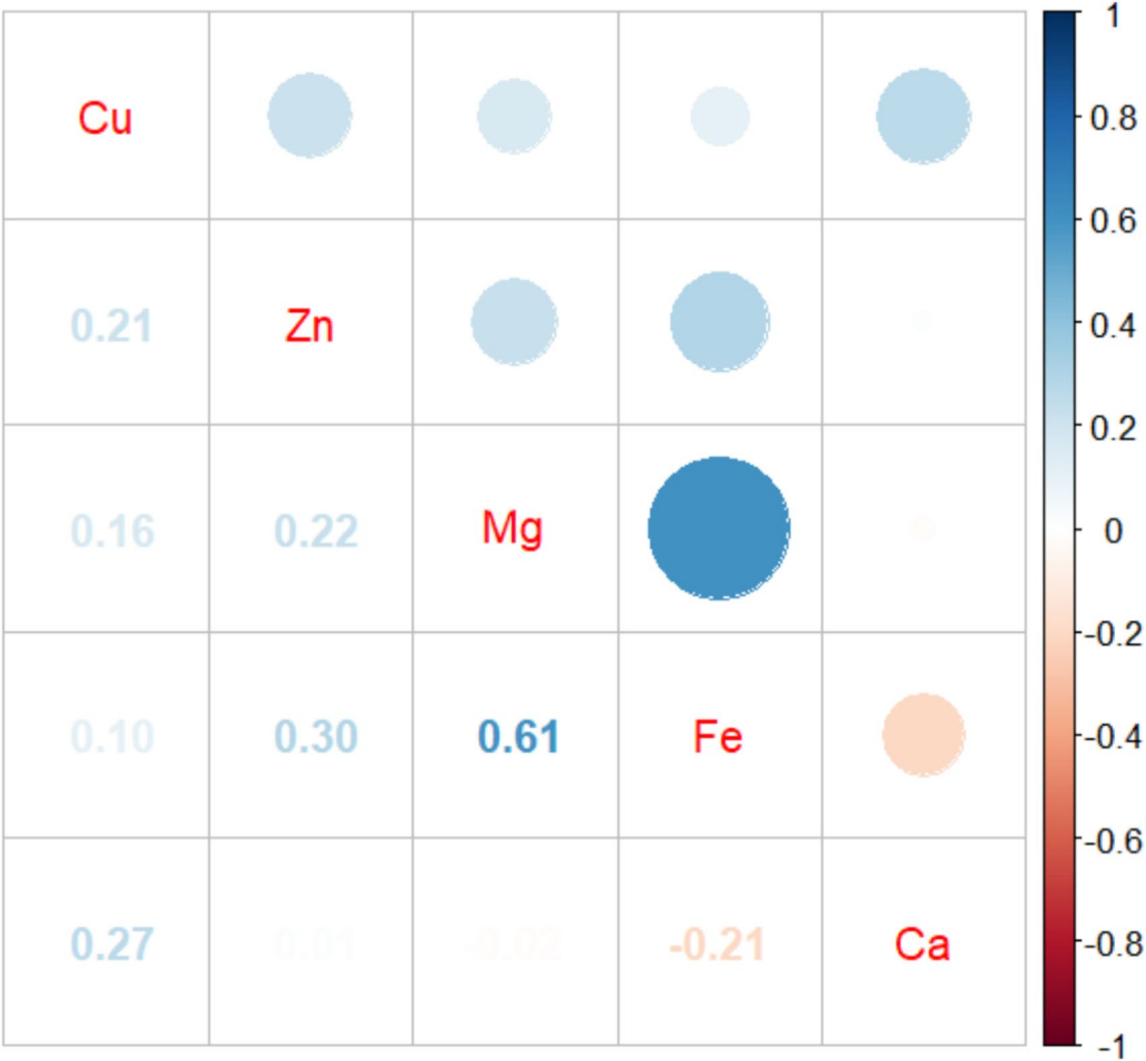
Pearson correlation analysis between five essential metal elements.

The results of Pearson correlation analysis between five essential metal elements were shown in Figure 2, and there was a positive correlation between most essential metal elements. Among them, Fe and Mg had the strongest correlation (*r* = 0.605, *p* < 0.001), and there was a negative correlation between Fe and Ca (*r* = −0.208, *p* < 0.001).

### 3.4 Regression models of GDM risk

#### 3.4.1 Correlation between individual essential metal element and GDM

The results of logistics regression were shown in Table 3. After adjusting the age, pre-pregnancy BMI, parity, detection season and gestational week of essential metal elements, Fe (*OR* = 1.171, 95%*CI*: 1.080 ~ 1.272), Zn (*OR* = 1.008, 95%*CI:* 1.004 ~ 1.013) and Mg (*OR* = 1.906, 95%*CI*: 1.237 ~ 2.937) levels showed positive correlations with GDM risk. However, no significant correlations were found between Ca or Cu levels and GDM (*p* > 0.05). Analysis by quartiles of essential metal elements revealed that higher quartile levels of Fe, Zn, and Mg were associated with increased GDM risk (*P_FDR_* < 0.05). Compared with the first quantile level, the fourth quantile level of Fe (*OR* = 1.347, 95%*CI*: 1.158 ~ 1.568), Zn (*OR* = 1.379, 95%*CI*: 1.185 ~ 1.606) and Mg (*OR* = 1.192, 95%*CI*: 1.022 ~ 1.392) were positively correlated with GDM. Upon further adjustment for other essential metal elements, only Zn maintained a positive correlation with GDM risk (*OR* = 1.006, 95%*CI*: 1.001 ~ 1.011). Notably, fourth quartile levels of Fe (*OR* = 1.221, 95%*CI*: 1.009 ~ 1.478) and Zn (*OR* = 1.272, 95%*CI*: 1.081 ~ 1.496) remained positively associated with GDM risk.

**Table 3:** Logistic regression analysis of the relationship between individual essential metal elements and GDM (*N* = 9,112).

Variables	Total(n)	GDM (*n*, %)	Model 1		Model 2		Model 3	
			OR (95% CI)	*P*	OR (95% CI)	*P*	OR (95% CI)	*P*
Fe (mmol/L)	9,112	1,697 (18.62)	**1.210 (1.118, 1.311)**	**<0.001**	**1.171 (1.080, 1.272)**	**<0.001**	1.104 (0.992, 1.230)	0.070
Quartile 1 (5.42 ~ 7.32)	2,270	380 (4.17)	Ref		Ref		Ref	
Quartile 2 (7.33 ~ 7.78)	2,276	401 (4.40)	1.064 (0.912, 1.241)	0.432	1.027 (0.877, 1.202)	0.742	1.008 (0.856, 1.186)	0.927
Quartile 3 (7.79 ~ 8.21)	2,280	405 (4.44)	1.074 (0.921, 1.253)	0.361	1.058 (0.904, 1.239)	0.480	1.008 (0.848, 1.199)	0.924
Quartile 4 (8.22 ~ 9.65)	2,286	511 (5.61)	**1.432 (1.236, 1.660)**	**<0.001**	**1.347 (1.158, 1.568)**	**<0.001**	**1.221 (1.009, 1.478)**	**0.041**
*P* for trend				**<0.001**		**<0.001**		**0.022**
Ca (mmol/L)	9,112	1,697 (18.62)	0.706 (0.420, 1.185)	0.189	0.878 (0.515, 1.496)	0.634	0.891 (0.502, 1.579)	0.693
Quartile 1 (1.25 ~ 1.48)	2066	398 (4.37)	Ref		Ref		Ref	
Quartile 2 (1.49 ~ 1.55)	2,402	444 (4.87)	0.950 (0.818, 1.105)	0.506	0.989 (0.848, 1.154)	0.889	1.001 (0.856, 1.170)	0.993
Quartile 3 (1.56 ~ 1.62)	2,308	425 (4.66)	0.946 (0.813, 1.101)	0.473	0.989 (0.847, 1.156)	0.891	1.004 (0.855, 1.179)	0.964
Quartile 4 (1.63 ~ 1.88)	2,336	430 (4.72)	0.945 (0.813, 1.100)	0.468	1.008 (0.864, 1.178)	0.915	1.024 (0.867, 1.208)	0.783
*P* for trend				0.496		0.904		0.759
Zn (μmol/L)	9,112	1,697 (18.62)	**1.010 (1.006, 1.015)**	**<0.001**	**1.008 (1.004, 1.013)**	**<0.001**	**1.006 (1.001, 1.011)**	**0.014**
Quartile 1 (46.30 ~ 76.60)	2,249	380 (4.06)	Ref		Ref		Ref	
Quartile 2 (76.61 ~ 84.10)	2,293	401 (4.43)	1.086 (0.930, 1.268)	0.296	1.100 (0.939, 1.288)	0.237	1.071 (0.913, 1.255)	0.399
Quartile 3 (84.11 ~ 92.10)	2,268	405 (4.47)	1.111 (0.952, 1.297)	0.184	1.091 (0.932, 1.278)	0.277	1.028 (0.874, 1.210)	0.737
Quartile 4 (92.11 ~ 121.90)	2,302	511 (5.66)	**1.467 (1.265, 1.703)**	**<0.001**	**1.379 (1.185, 1.606)**	**<0.001**	**1.272 (1.081, 1.496)**	**0.004**
*P* for trend				**<0.001**		**<0.001**		**0.005**
Cu (μmol/L)	9,112	1,697 (18.62)	**1.016 (1.004, 1.028)**	**0.007**	1.012 (0.999, 1.024)	0.051	1.007 (0.994, 1.020)	0.305
Quartile 1 (10.10 ~ 19.70)	2,266	406 (4.45)	Ref		Ref		Ref	
Quartile 2 (19.71 ~ 22.70)	2,175	370 (4.06)	0.939 (0.804, 1.097)	0.427	0.913 (0.779, 1.070)	0.264	0.901 (0.768, 1.057)	0.202
Quartile 3 (22.71 ~ 26.00)	2,362	454 (4.98)	1.090 (0.940, 1.265)	0.254	1.048 (0.900, 1.221)	0.543	1.013 (0.867, 1.184)	0.869
Quartile 4 (26.01 ~ 38.40)	2,309	467 (5.13)	**1.161 (1.002, 1.3477)**	**0.047**	1.107 (0.951, 1.290)	0.190	1.044 (0.887, 1.229)	0.603
*P* for trend				**0.013**		0.073		0.438
Mg (mmol/L)	9,112	1,697 (18.62)	**2.575 (1.693, 3.916)**	**<0.001**	**1.906 (1.237, 2.937)**	**0.003**	1.169 (0.676, 2.020)	0.577
Quartile 1 (0.98 ~ 1.30)	2,167	360 (3.95)	Ref		Ref		Ref	
Quartile 2 (1.31 ~ 1.38)	2,243	403 (4.42)	1.099 (0.940, 1.286)	0.235	1.035 (0.883, 1.215)	0.670	0.972 (0.823, 1.147)	0.735
Quartile 3 (1.39 ~ 1.47)	2,329	439 (4.82)	1.165 (0.999, 1.360)	0.051	1.092 (0.934, 1.278)	0.270	0.986 (0.831, 1.171)	0.873
Quartile 4 (1.48 ~ 1.73)	2,373	495 (5.43)	**1.323 (1.139, 1.538)**	**<0.001**	**1.192 (1.022, 1.392)**	**0.026**	0.998 (0.827, 1.205)	0.982
*P* for trend				**<0.001**		**0.017**		0.995

Ref, reference group; OR, odds ratio; CI, confidence interval; GDM, gestational diabetes mellitus.

Model 1: not adjusted any variables.

Model 2: adjusted the age of pregnant women, pre-pregnancy BMI, parity, season and gestational week at blood specimen collection.

Model 3: adjusted for all variables in Model 2 and other four trace metals (continuous variables).

*P* for trend was obtained from the conditional logistic regression models by using the median of each quartile of essential metal element concentrations as continuous variables.

The bold black font indicates that the association is statistically significant (*P* < 0.05).

#### 3.4.2 Exposure-response relationship between individual essential metal element and GDM

**Figure 3:**
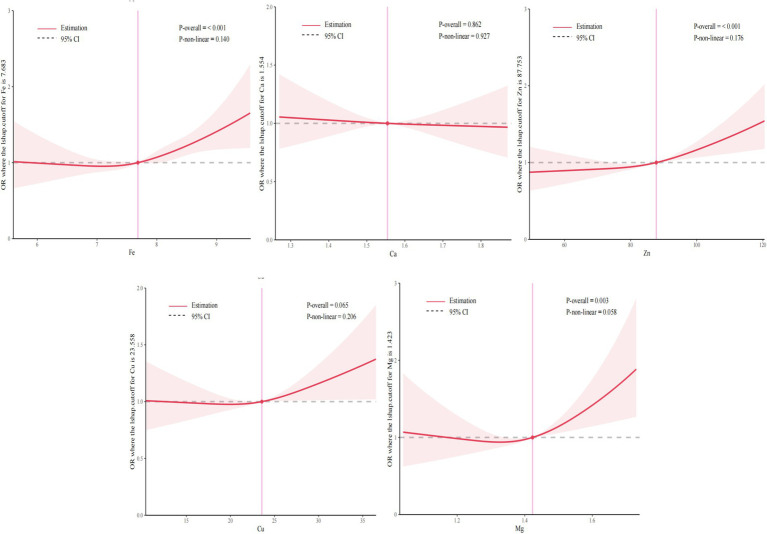
Exposure-reaction relationship between five essential metal elements and GDM. The model adjusted the age of pregnant women, pre-pregnancy BMI, parity, season and gestational week at blood specimen collection.

Furthermore, Figure 3 illustrated the results of RCS analysis. After adjusting for covariates, a positive linear relationship was observed between Fe, Zn, and Mg levels and the risk of GDM (*P_non-linear_* > 0.05 and *P_non-linear_* > 0.05). Among them, the inflection point of Fe was 7.68 mmol/L, Zn was 87.75 μmol/L and Mg was 1.42 mmol/L. There was no significant correlation between Ca and Cu levels and the risk of GDM (*P_overall_* > 0.05 and *P_non-linear_* > 0.05).

#### 3.4.3 Correlation between multiple essential metal elements and GDM

**Figure 4:**
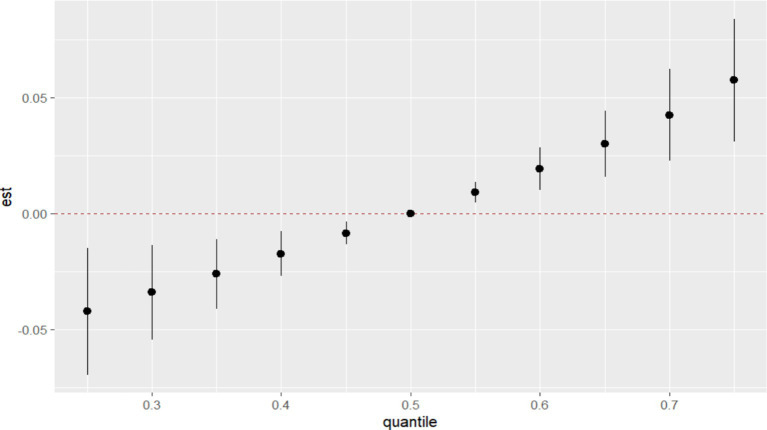
Combined effect of essential metal elements on GDM.

**Figure 5:**
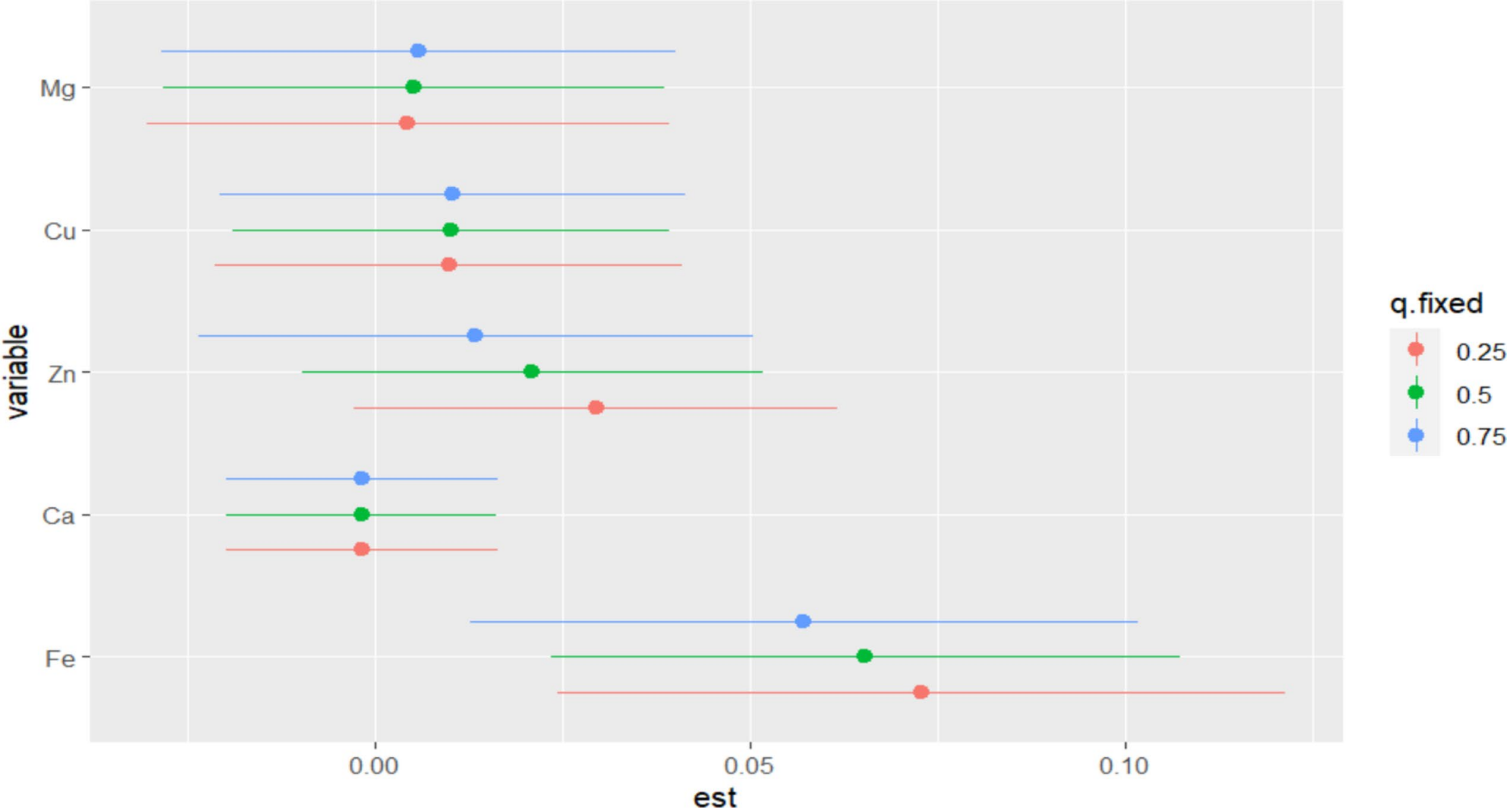
Individual effects of essential metal elements on GDM correlation.

**Figure 6:**
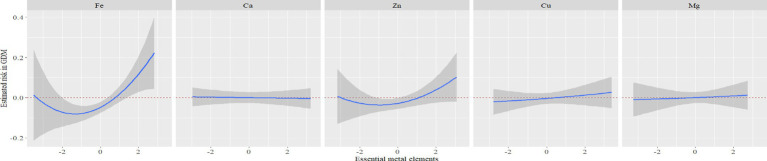
Exposure-response relationship between individual essential metal elements and GDM.

**Figure 7:**
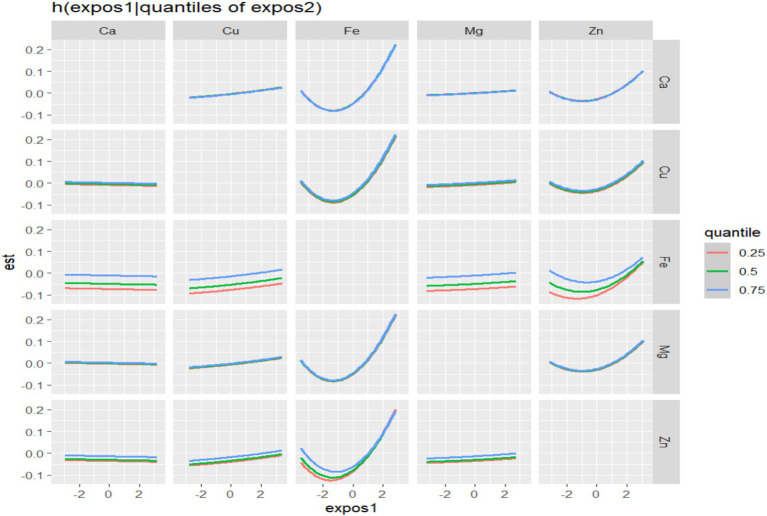
Bivariate exposure-effect curve of essential metal elements associated with GDM.

In BKMR, the PIP values of the essential metal elements were shown in Table 4, and Fe (PIP = 0.772) had the greatest influence on the combined action. The combined effects of five essential metal elements on GDM were shown in Figure 4, and the combined effects of all essential metal elements on GDM generally showed an upward trend. The individual effect of a single essential metal element on GDM risk was shown in Figure 5, in which Fe played a key role in the combined effect of essential metal elements and GDM. In addition, the exposure-response relationship between each essential metal element and GDM was shown in Figure 6, and the effects of Fe and Zn on GDM showed a trend of first decreasing and then increasing, which was more in line with the non-linear correlation. No obvious interaction between essential metal elements was found in this study, and the results were shown in Figure 7.

**Table 4:** PIP of each essential metal elements in BKMR (*N* = 9,112).

Variables	PIP
Fe	0.772
Ca	<0.001
Zn	0.166
Cu	0.007
Mg	0.005

The model adjusted the age of pregnant women, pre-pregnancy BMI, parity, season and gestational week at blood specimen collection.

### 3.5 Sensitivity analyses

Supplementary Tables S1, S2 presents the results of WQS and QGC. After covariate correction, the combined action amount of five essential metal elements and the risk of GDM showed a monotonically increasing positive relationship, in which Fe had a larger positive weight in the relationship between the mixture and GDM (Supplementary Figures S1, S2).

All the above analysis results showed that Fe played an important role in GDM, and BKMR model had not found any interaction between essential metal elements on GDM, so this study further used multiplication interaction and addition interaction to verify whether Fe and other essential metal elements interact with GDM. The multiplication interaction results were shown in Supplementary Table S3 and the addition interaction results were shown in Supplementary Table S4. After adjusting the covariate, there was still no multiplication or addition interaction between the essential metal elements and GDM.

## 4 Discussion

The occurrence of GDM was a complex process, and its pathogenesis was still not fully clarified. Previous studies have shown heterogeneity in the relationship between different levels of essential metals. This study provided reliable results to explore the influence of essential metal elements in early pregnancy on GDM risk.

Fe was one of the important trace essential metal elements in human body (51). The results from the BKMR and GLM model indicated Fe were associated with an increased risk of GDM. The negative correlation found between Ca and GDM in the BKMR model might offset the positive correlations observed between other essential metal elements and GDM to some extent (52). This underscored the importance of combining classical single exposure effect models with mixed exposure effect models (53). On the other hand, it was emphasized that Fe was associated with an increased risk of GDM in the comprehensive effect. A study using data from the Ma’anshan birth cohort (MABC) recruited 3,289 pregnant women. Their results showed that the risk of GDM in pregnant women with the highest level of serum Fe was 1.63 times higher than that in the middle level (54). The Wuhan twin birth cohort (WTBC) study from 2013 to 2016 also found that the risk of GDM in pregnant women with serum Fe concentration in the fourth quartile was 5.13 times higher than that in the first quartile (55). Elevated Fe levels may contribute to increased oxidative stress, potentially leading to damage to pancreatic β cells, reduced insulin secretion, and impaired glucose regulation (56). High levels of Fe increased the burden on the liver, reduced the sensitivity of the liver to insulin, induced insulin resistance, reduced the synthesis of liver glycogen and weakened the sensitivity to insulin signals (57). High levels of Fe can also reduce the supply of glucose oxidation energy and enhance fatty acid metabolism in skeletal muscle cells, reduce insulin-induced glucose transport in adipocytes, and ultimately lead to GDM (58).

As an auxiliary factor of many enzymes, Zn was second only to Fe in the whole-body content (59). Clinical and animal studies have shown that Zn can directly regulate insulin activity and glucose homeostasis (60). More and more evidence was consistent with the results of this study, supporting the relationship between Zn and GDM (61, 62). The results of this study showed that the risk of GDM in pregnant women with Zn at the fourth quantile level (92.10 ~ 121.90 *μ* mol/L) was 1.379 times that at the lowest quantile level. The mechanism between high levels of Zn and GDM includes the direct effects of Zn on oxidative stress, immune regulation and insulin activity (63). In addition, Zn has estrogen activity (64), which can simulate some characteristics of insulin secreted by pancreatic β cells and interfere with insulin metabolism and glucose homeostasis, thus increasing the risk of GDM (65). However, the correlation between Zn and GDM in some previous studies was not consistent with the results of this study. A previous case–control study in Wuhan, China showed that compared with the lowest quartile level, the risk of GDM in pregnant women with Zn at the highest quartile level in the first trimester was 0.30 (95%*CI*: 0.18 ~ 0.50) (55). Zhang and Liang (66) recruited pregnant women who received obstetric examination in the obstetric clinic of Shanxi Provincial People’s Hospital as the research objects, and found that there was no correlation between Zn level and pregnant women’s blood sugar level. A prospective cohort study of pregnant women in Australia by Wilson et al. (67) also showed that Zn level in early pregnancy was not related to GDM.

Mg, involved in various enzymatic reactions in the body (68), affecting glucose metabolism stability and insulin sensitivity (69). Impairment of Mg homeostasis at insulin receptor will lead to insulin resistance and decrease of insulin secretion in β cells, leading to an increase in GDM risk (70). The results of this study showed that the higher level of Mg was positively correlated with the risk of GDM (*OR* = 1.192, 95%*CI*: 1.022 ~ 1.392), and the risk of GDM increased significantly when the blood Mg level was greater than 1.42 mmol/L. The results were similar to a prospective cohort study with 65 pregnant women, indicating that the risk of GDM increased 3.923 times per unit level of Mg (66). However, a previous case–control study included 610 pregnant women as the research object, and the study showed that there was no significant correlation between Mg and GDM (55). Differences in sample size might explain the discrepancies in the findings.

In addition to the main findings, our research found no correlation between Cu, calcium and GDM. However, studies have shown that there was a negative correlation between Ca and GDM in early pregnancy (*RR* = 0.84, 95%*CI*: 0.73, 0.97), and a positive correlation between Cu and GDM (*RR* = 1.23, 95%*CI*: 1.02, 1.49) (55). Although the essential metal elements examined in their study are consistent with our research, differences detection methods for essential metal elements, and covariates may contribute to the discrepancies in the findings. Ca homeostasis affected insulin secretion and the survival of β cells, while calcium channel blockers had shown efficacy in preventing GDM (71). Excessive Cu would increase oxidative stress, leading to β cell death and glucose metabolism disorder (72). Future studies with larger sample sizes, more refined measurement techniques, and careful control of confounding factors may provide a clearer understanding of these trace elements’ role in GDM.

This study suggested that while essential for normal metabolic function, the combined action of these five elements may also elevate the risk of GDM. Some studies supported the results of this study (73–75). Li et al. (76) used BKMR model to evaluate the correlation between FPG and the combined effects of five elements, including urinary arsenic (As), nickel (Ni), cadmium (Cd) and plasma selenium (Se) and Zn. The results showed that these five elements were also positively associated with FPG (76). However, in this study, besides Se and Zn, three harmful elements, AS, Ni and Cd, were also included in the overall effect evaluation. The biological samples used in the above study were also different from this study, which used urine and plasma samples to detect elements, while this study used whole blood samples. A retrospective cohort study included 8,169 pregnant women in China, which proved that the WQS index of essential metal elements such as manganese (Mn), Cu, lead (Pb), Ca, Zn and Mg was significantly positively correlated with FBG (26). Among the six essential metal elements included in the above study, Mn was the most important in the combined effect. The difference of results may be related to the types of metal elements and the mechanism between essential metals and GDM was not clear.

Previous studies have shown that different essential metal elements may have synergistic or antagonistic interactions on health effects, particularly in relation to pregnancy and maternal health (77). Zheng et al. (78) evaluated the relationship between Zn, Se, Cu and molybdenum as a mixture and the glucose level in pregnancy in a multi-ethnic pregnancy cohort, and found that the synergistic effect of Cu and Zn would increase the glucose level during pregnancy (78). It may be related to the synergistic activation of Cu/Zn superoxide dismutase by Cu and Zn (79). Cu/Zn superoxide dismutase promotes the production of ROS by acting as superoxide reductase or superoxide oxidase, which leads to metabolic disorders and affects glucose levels (80). Nested case–control study in pregnant women in China also showed that Ca and Fe had significant synergistic effect on GDM (55). In addition, Mg and Ca had a strong synergistic effect, which affected FPG and increases the risk of GDM (81). The synergistic effect of Mg and Ca may be related to the secretion of parathyroid hormone. Thyroid function can mediate the relationship between essential metal elements and GDM (66). The interaction mode of essential metals was influenced by dose level, dose ratio or mixture composition (82). Therefore, it was still necessary to explain the interaction between essential metal elements (83).

It was worth noting that with the development of economy, pregnant women pay more attention to the supplement of nutrients during pregnancy, and whether healthy pregnant women need regular supplement of essential metal elements needs further evaluation. Previous studies have shown that Fe supplementation for pregnant women without Fe deficiency anemia in the first trimester is positively correlated with the increase of hemoglobin level and blood sugar level, and the risk of GDM also increases (84). High levels of Fe can also produce reactive oxygen species (ROS) and cause tissue damage, leading to premature delivery, low birth weight infants and neurodevelopmental defects in children and other adverse pregnancy outcomes (85, 86). Zinc played a crucial and direct role in the development of the fetal nervous system and the prevention of pregnancy complications (87). Additionally, studies suggested that magnesium reduced oxidative stress and helped prevent complications like pregnancy-induced hypertension and preeclampsia (88). The WHO suggested that prenatal health care should promote positive pregnancy experience, and the population size, distribution and Fe deficiency of anemia should be fully considered under strict research background before Fe supplements can be recommended (89). Zinc and Mg supplements were not recommended as part of routine pregnancy care, but only in the context of rigorous research (90). Essential metal element detection should be included in routine prenatal exams, as early detection is crucial to prevent imbalances that could harm both mother and fetus. Additionally, mobile health tools (mHealth) were used to monitor various health indicators in pregnant women with GDM and played a key role in improving their dietary compliance and behavior changes (91). In the future, greater attention should be given to this area.

## 5 Limitations

Single center design was one of the main limitations of this study. This study was observational and only identified an association between essential metal elements and GDM, without determining a causal relationship. At the same time, this study did not collect the information of pregnant women’s dietary intake, dietary supplements intake, atmospheric environment during pregnancy and household fuel use. These factors were closely related to the level of essential metal elements, and related research should be further included in the above factors in the future. Only five essential metal elements, such as Fe, Cu, Zn, Ca and Mg, were included in the assessment of combined effect. Pregnant women may be exposed to a wider range of environment in the real world, and indicators of other essential metal elements need to be collected.

## 6 Conclusion

The research findings unveiled a notable association between essential metal elements and GDM. Particularly, higher concentrations of Fe, Zn, and Mg manifested a significant positive correlation with GDM, emphasizing the criticality of adequate intake of these elements during pregnancy. The combined impact of these elements manifested a monotonically increasing relationship with the risk of GDM, with Fe exerting the most substantial influence, suggesting Fe as a potential biomarker for GDM risk. However, no significant interactions between the essential metal elements and GDM were observed. Given the complexity of trace element balance during pregnancy, further research is needed to investigate their collective impact on GDM development.

## Data Availability

The raw data supporting the conclusions of this article will be made available by the authors, without undue reservation.
