# Supplementary material for: Association between blood essential metal elements in early pregnancy and gestational diabetes mellitus
Source: Front Nutr. 2025 Jun 19;12:1554840. doi: 10.3389/fnut.2025.1554840 (PMC12222152; doi:10.3389/fnut.2025.1554840)
Supplement: Supplementary file 1 [file Supplementary_file_1.pdf]

Table S1. the relationship between multiple essential metal elements and GDM in WQS (N = 9112)

	OR (95% *CI*)	*P*	weight proportion				
			Fe	Ca	Zn	Cu	Mg
Model 1	**1.120 (1.092, 1.318)**	**< 0.001**	0.382	0.145	0.360	0.074	0.039
Model 2	**1.129 (1.023, 1.247)**	**0.016**	0.446	0.218	0.283	0.044	0.009

Model 1: not adjusted any variables.

Model 2: adjusted the age of pregnant women, pre-pregnancy BMI, parity, season and gestational week at blood specimen collection.

The bold black font indicates that the association is statistically significant (P < 0.05).

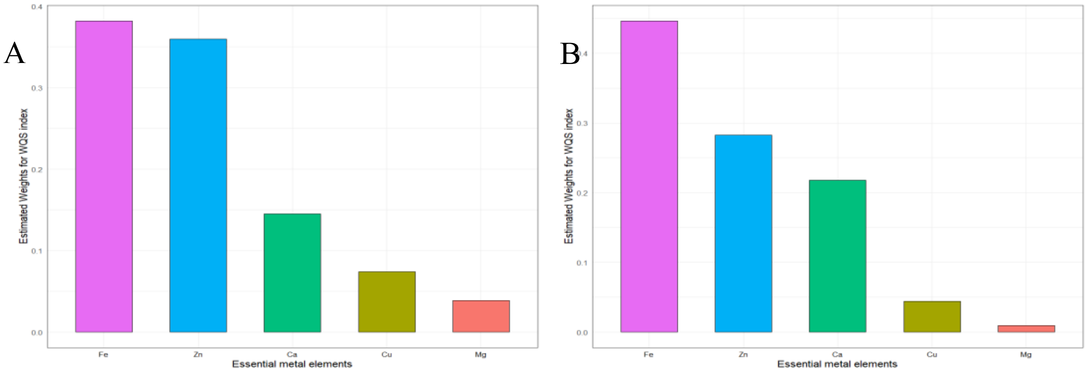

Fig. S1. Estimated weight distribution of each essential metal element in the association in WQS

Fig. A: not adjusted any variables.

Fig. B: adjusted the age of pregnant women, pre-pregnancy BMI, parity, season and gestational week at blood specimen collection.

Table S2. the relationship between multiple essential metal elements and GDM in QGC (N = 9112)

	OR (95% *CI*)	*P*	weight proportion				
			Fe	Ca	Zn	Cu	Mg
Model 1	**1.189 (1.104, 1.274)**	**< 0.001**	0.268	-1.000	0.423	0.170	0.139
Model 2	**1.161 (1.075, 1.248)**	**< 0.001**	0.417	-0.516	0.454	0.129	-0.484

Model 1: not adjusted any variables.

Model 2: adjusted the age of pregnant women, pre-pregnancy BMI, parity, season and gestational week at blood specimen collection.

The bold black font indicates that the association is statistically significant (P < 0.05).

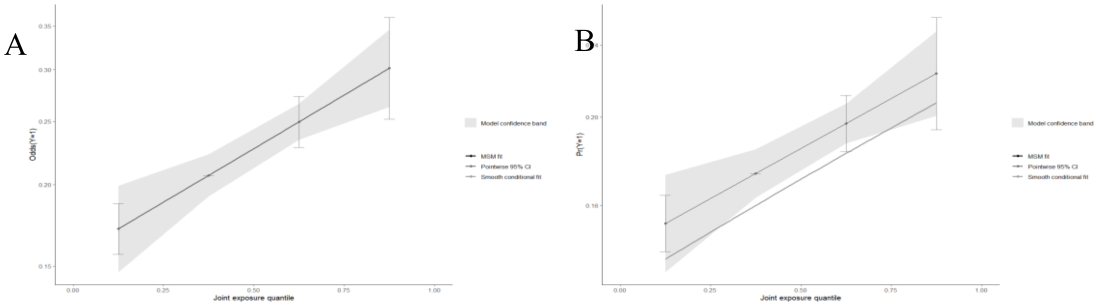

Fig. S2. Linear trend of risk influence of multiple essential metal elements on GDM in QGC

Figure A: not adjusted any variables.

Figure B: adjusted the age of pregnant women, pre-pregnancy BMI, parity, season and gestational week at blood specimen collection.

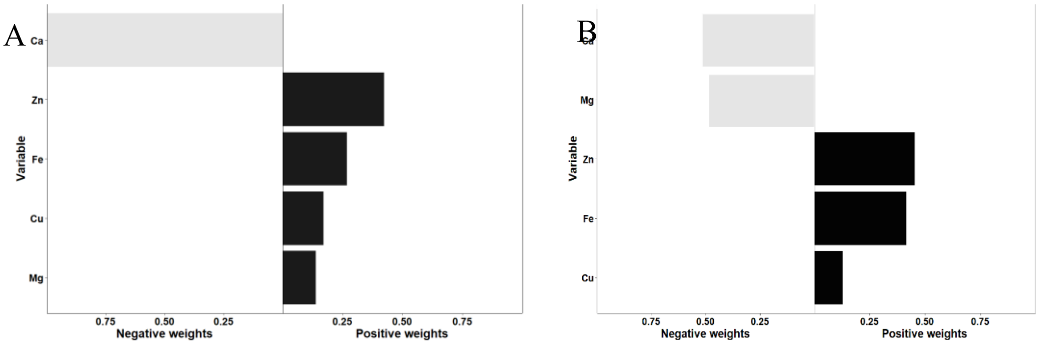

Fig. S3. Correlation weight distribution of QGC index and GDM

Figure A: not adjusted any variables.

Figure B: adjusted the age of pregnant women, pre-pregnancy BMI, parity, season and gestational week at blood specimen collection.

Table S3. multiplication interaction of Fe and other essential metal elements and GDM (N=9112)

variables	Model 1		Model 2	
	OR (95% *CI*)	*P*	*OR* (95% *CI*)	*P*
Fe and Ca	1.097 (0.812, 1.479)	0.546	1.088 (0.800, 1.477)	0.590
Fe and Zn	1.006 (0.739, 1.375)	0.967	1.001 (0.729, 1.375)	0.999
Fe and Cu	0.964 (0.716, 1.299)	0.809	0.969 (0.714, 1.315)	0.839
Fe and Mg	1.266 (0.906, 1.781)	0.171	1.231 (0.874, 1.745)	0.238

Model 1: not adjusted any variables.

Model 2: adjusted the age of pregnant women, pre-pregnancy BMI, parity, season and gestational week at blood specimen collection.

Table S4. additive interaction between iron and other essential metal elements and GDM (N=9112)

variables	Model 1		Model 2	
	estimated value	95% *CI*	estimated value	95% *CI*
Fe and Ca				
RERI	-0.096	(-0.435, 0.243)	0.121	(0.034, 0.207)
AP	-0.082	(-0.371, 0.207)	0.091	(0.057, 0.125)
S	0.642	(0.166, 2.473)	1.607	(0.951, 2.715)
Fe and Zn				
RERI	0.021	(-0.329, 0.371)	0.122	(0.029, 0.214)
AP	0.016	(-0.255, 0.288)	0.091	(0.054, 0.128)
S	1.078	(0.287, 4.047)	1.574	(0.984, 2.516)
Fe and Cu				
RERI	-0.031	(-0.364, 0.302)	0.118	(0.035, 0.201)
AP	-0.026	(-0.301, 0.250)	0.091	(0.059, 0.123)
S	0.871	(0.217, 3.497)	1.664	(0.859, 3.223)

Fe and Mg				
RERI	-0.264	(-0.662, 0.134)	0.078	(0.048, 0.107)
AP	-0.237	(-0.608, 0.133)	0.084	(0.037, 0.131)
S	0.296	(0.027, 3.186)	0.484	(0.036, 6.562)

Model 1: not adjusted any variables.

Model 2: adjusted the age of pregnant women, pre-pregnancy BMI, parity, season and gestational week at blood specimen collection.
